# Post-processing enhances protein secondary structure prediction with second order deep learning and embeddings

**DOI:** 10.1016/j.csbj.2024.12.022

**Published:** 2025-01-02

**Authors:** Sotiris Chatzimiltis, Michalis Agathocleous, Vasilis J. Promponas, Chris Christodoulou

**Affiliations:** a University of Cyprus, Department of Computer Science, Nicosia, Cyprus; b University of Nicosia, Department of Computer Science, Nicosia, Cyprus; c University of Cyprus, Department of Biological Sciences, Nicosia, Cyprus; d 5G/6GIC, Institute for Communication Systems (ICS), University of Surrey, Guildford, United Kingdom

**Keywords:** 0000, 1111, Convolutional neural networks, Deep learning, Embeddings, Hessian free optimisation, Protein secondary structure prediction

## Abstract

Protein Secondary Structure Prediction (PSSP) is regarded as a challenging task in bioinformatics, and numerous approaches to achieve a more accurate prediction have been proposed. Accurate PSSP can be instrumental in inferring protein tertiary structure and their functions. Machine Learning and in particular Deep Learning approaches show promising results for the PSSP problem. In this paper, we deploy a Convolutional Neural Network (CNN) trained with the Subsampled Hessian Newton (SHN) method (a Hessian Free Optimisation variant), with a two- dimensional input representation of embeddings extracted from a language model pretrained with protein sequences. Utilising a CNN trained with the SHN method and the input embeddings, we achieved on average a 79.96% per residue (Q3) accuracy on the CB513 dataset and 81.45% Q3 accuracy on the PISCES dataset (without any post-processing techniques applied). The application of ensembles and filtering techniques to the results of the CNN improved the overall prediction performance. The Q3 accuracy on the CB513 increased to 93.65% and for the PISCES dataset to 87.13%. Moreover, our method was evaluated using the CASP13 dataset where we showed that as the post-processing window size increased, the prediction performance increased as well. In fact, with the biggest post-processing window size (limited by the smallest CASP13 protein), we achieved a Q3 accuracy of 98.12% and a Segment Overlap (SOV) score of 96.98 on the CASP13 dataset when the CNNs were trained with the PISCES dataset. Finally, we showed that input representations from embeddings can perform equally well as representations extracted from multiple sequence alignments.

## Graphical abstract

**Figure:**
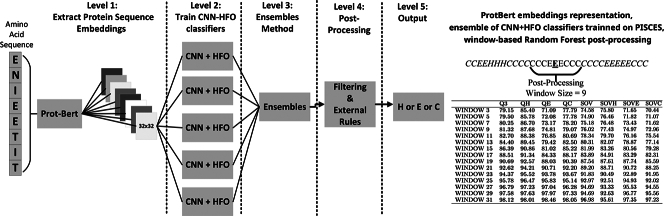

## 1 Introduction

Proteins are highly complex substances, present in all living organisms, that directly contribute to the chemical processes essential for life. In order to explain the function of a protein we need to know how amino acids composing it interact and fold in a three-dimensional (3D) space. Traditionally, protein structure is described using a 4-levelled hierarchical approach. The primary structure is the linear sequence of amino acids that form a specific protein. Secondary structure refers to the local folding of one polypeptide chain of a protein due to hydrogen bonds that are formed between the backbone-chain peptide groups. The tertiary structure is a three-dimensional (3D) representation of the polypeptide chain, which determines the specific function of a protein. Finally, the quaternary structure is the structure of a protein macromolecule formed by interactions of multiple polypeptide chains [1].

At the moment there are millions of proteins with known primary structure, but only for a small fraction of them we know their secondary and tertiary structure. The reason is that experimental methods used to determine secondary and tertiary structures are time-consuming and expensive [2], [3], [4], [5], [6]. Thus, this process is a bottleneck of the pipeline for the protein structure/function determination. There exists, therefore, an emerging need to develop methods for predicting the secondary and tertiary structures, which are considerably cheaper and require less time than experimental methods.

Predicting the secondary structure of proteins is a problem that researchers have been working on for more than six decades [7]. Multiple machine learning algorithms (including deep learning) have been developed over the years that are dedicated to this problem [8], [9], [10], [11], [12], [13], [14], [15], [16], [17], [18], [19], [20], [21], [22], [23], [24]. Algorithms using only machine learning techniques have reported three-state accuracy of around 85% whereas algorithms that rely on sequence-based structural similarities have achieved Q3 accuracy over 90% [25]. Despite the remarkable advancements made by AlphaFold and similar deep learning-based methods in predicting protein structures in atomic detail [26], [27], accurate secondary structure prediction methods can still prove of value for several reasons. First, they can provide information about the local structural features of proteins, which can lead to a more comprehensive understanding of protein structure, but also to structural dynamics: such a case could involve regions predicted with low confidence by structure prediction methods but are predicted to adopt different secondary structural states. In addition, PSSP methods are typically of lower computational complexity, thus more efficient for practical applications and are traditionally applied in sequence-based bioinformatics workflows.

In this paper we present an approach which can be classified in the post-AF2 category of deep learning for PSSP techniques, according to a recent review [28]. Specifically, we employ a convolutional neural network (CNN) trained with a variation of the Hessian Free Optimisation (HFO) algorithm [29], [30], known as the Subsampled Hessian Newton (SHN) method [31], to predict the secondary structure of proteins, using embeddings (as inputs to the CNN) extracted from the Bidirectional Encoder Representations (BERT) language model [32] based on protein primary structure. This CNN-SHN combination was previously used by Leontiou [33] as an attempt to solve the PSSP problem, while the HFO algorithm with simple Feed Forward Neural Networks was recently employed by Charalambous et al. [34] on the same problem. Moreover, in this paper we utilize post-processing techniques [35] that try to improve the overall prediction accuracy of the secondary structure predicted by the CNN. The implementation is available as a Jupyter notebook at https://gitlab.com/schatz06/pssp/-/tree/master/.

## 2 Materials and methods

### 2.1 Datasets

High quality datasets are essential for training and validating useful prediction models. To demonstrate the capabilities of CNN-SHN, we employed three benchmark datasets, CB513 [8], PISCES [36] and CASP13 ($13^{th}$ Critical Assessment of Protein Structure), which have been widely used in previous PSSP studies, each serving a different purpose. The CB513 dataset contains 513 unique proteins with maximum similarity per protein pair of 25%. The CB513 dataset was mainly used for tuning the hyper-parameters of the network. CB513 was split into ten folds which were used to train different CNN models using the cross-validation technique. The PISCES dataset contains 8632 protein sequences with also maximum similarity per protein 25% and a high-quality experimentally determined structure (resolution cutoff of 3.0 Å, and an R-factor cutoff of 1.0). A larger dataset like PISCES can help the model to learn more effectively the patterns of the data, resulting in higher performance. PISCES was split into five distinct folds as described in [37], instead of ten mainly because the required training time for a fold was much larger compared to a CB513 fold. Finally, the CASP13 dataset was used for testing which contains 40 protein sequences. The CASP13 dataset contains 3 protein sequences that have similarity with at least one protein in the CB513 dataset. Furthermore, in CASP13 13 out of 40 sequences matched at least one protein in the PISCES dataset. Leontiou et al. [33] produced results both on the full CASP13 dataset and the reduced CASP13 dataset (with the matching proteins removed) when their models were trained with the PISCES dataset, and they showed a minor change in performance. Since there was no significant fluctuation on their results, in this paper we decided not to exclude any protein sequences from the CASP13 dataset.

It is worth mentioning that there exist numerous examples in the published literature where homologous proteins (usually from phyogenetically diverse species) can exhibit much lower than 25% identity (see e.g., [38], [39]). However, using a more stringent threshold for eliminating sequence redundancy is expected to remove a non-negligible fraction of sequence data for both training and validating/testing our models, thus we decided to go with the 25% threshold which is widely used in similar applications.

### 2.2 Convolutional neural networks

A CNN is a class of deep artificial neural networks, which is most commonly applied to analyze visual imagery [40]. CNNs have a wide range of applications, from image and video recognition, recommender systems, image classification to medical image analysis, and Natural Language Processing (NLP). CNNs are an extension of traditional Multilayer Perceptons (MLPs), which perform convolution instead of matrix multiplication in at least one of their layers.

CNNs consist of an input and an output layer, and multiple hidden layers. Those hidden layers are classified into convolutional layers and sub-sampling (pooling) layers. The activation function used is Rectified Linear Unit (RELU) and it is applied after a convolutional layer. CNNs end with a fully connected layer, usually an MLP network. The input of a convolutional neural network is an image of size d × d × c, where d is the height and width of the image and c represents the number of channels the input image has (e.g., for RGB, Red Green Blue, c = 3; for GRAYSCALE c = 1). A convolutional layer has n filters (kernels) of size k × k × m, where k is smaller than the dimensions of the image and m can be either the same as the number of channels (c) or smaller. Convolutional layers convolve the input, which leads to n feature maps of size smaller or equal to d – k + 1 and pass their output to the next layer. If the next layer is a convolutional layer the same process is repeated, but if the next layer is a pooling layer, the feature maps are sub-sampled, typically averaging or maximizing above the same areas in feature maps of size p × p (where p is between 2 and 5 depending on the size of the image). Pooling layers are typically placed between two convolutional layers. The main scope of pooling layers is to reduce the dimensions of the feature maps produced by convolutional layers. Reducing the dimension of a feature map reduces the number of parameters and the complexity of the network, which consequently reduces the total computation time of the network, as well as prevents the network from overfitting [41]. However, using sub-sampled layers may remove details that are useful to train the network resulting in poorer overall performance. There are several types of pooling layers such as min pooling, max pooling, average pooling and L2-normalization pooling. Finally, in some cases we may need to apply padding around the image in order to control the dimensions of the outputs produced by the convolution layers. There are several types of padding such as constant padding and zero padding.

### 2.3 Data representation method

This paper employs embeddings as input to the CNN. Those embeddings were extracted from the ProtBert model [42], [43]. The current model is basically the BERT (Bidirectional Encoder Representations from Transformers) [32] language model but adapted to extract protein embeddings. BERT is the current state of the art NLP framework. BERT is designed to pre-train deep bidirectional representations from unlabelled text by jointly conditioning on both left and right context [32]. BERT is based on the encoder part of Transformers. Transformers are the first transduction model relying entirely on self-attention to compute representations of its input and output without using sequence-aligned Recurrent Neural Networks (RNNs) or convolution. Self-attention, also called intra-attention, is a mechanism that relates different positions of a single sequence in order to compute a representation of the sequence [44].

ProtBert, as mentioned before, is simply the BERT model trained on a large corpus of unlabelled protein sequence data. There are two different versions of the ProtBert embedder, the first version was trained with the UniREF100 dataset that contained 216 million protein sequences, and the second with the BFD dataset that has 2100 million protein sequences [42]. This paper makes use of the version trained with the BFD dataset with the mindset that an embedder that is trained with more data will extract more powerful embeddings. The main concept behind this approach is to interpret protein sequences as sentences and every amino acid in a protein sequence as a word. The secondary structure of every amino acid in a protein sequence depends on the values of its neighbouring amino acids. ProtBert uses all the preceding and succeeding amino acids to create a contextual representation of a single amino acid in every protein sequence, thus helping in the secondary structure prediction.

During the training phase of the ProtBert model useful features and constraints are extracted from protein sequences. The vector from the last hidden state of the transformer's attention stack was captured to be used as input for secondary structure prediction. Thus, each amino acid residue of a protein sequence is represented with a vector of size 1024, which is then transformed into a 32 × 32 × 1 matrix in order to be used as input to the CNN.

### 2.4 Proposed convolutional neural network architecture with subsampled Hessian Newton method

In this paper we use CNN with the SHN method, which is a variation of HFO. The CNN consists of four active layers, with the first three layers being convolutional layers (Conv2D). The first two convolutional layers have 64 filters of size 5 × 5 × 1 each, and the last convolutional layer has 128 filters of shape 5 × 5 × 1. The size of each feature map, that is created after applying each of the 64 filters, is the same as the input size (32 × 32 × 1). To ensure that the shape of the output of each convolutional layer remains the same as the input shape, padding of zeroes is applied around the input data before applying the convolution. Pooling layers were not utilized in this CNN architecture since they removed sufficient information resulting in a poorer model performance. All three convolutional layers use the Rectifier Linear Unit (RELU) as activation function. The last layer of the CNN is a fully connected (Dense) layer, which consists of three neurons. The output from this layer is an array with three values, which are used to classify the flattened feature maps to one of the three secondary structure classes (C, E, H), using the SoftMax function. The CNN was trained for up to 100 epochs, using the mean square error (MSE) as loss function, utilizing the SHN optimization method (see next).

Several studies were undertaken that train artificial neural networks (ANNs) using Newton's methods [29], [45], [46]. However, most of them utilized only fully connected feed forward neural networks (FFNNs). This made Gradient Descent and its variants the default optimisation algorithms for deep neural networks. Wang et al. [31], theoretically developed the application of Newton methods for CNNs. An example can be the HFO optimisation algorithm, which has been shown to outperform the backpropagation algorithm in terms of performance (accuracy, convergence rate) and can successfully tackle the vanishing-gradient problem. HFO computes an approximation of the Hessian Matrix and employs the Conjugate Gradient algorithm to compute and update the network's weights. Furthermore Wang et al. [31] proposed a new optimization method that reduces the computation intensity of approximating the Hessian Matrix, the SHN method. SHN uses the subsampled Gauss-Newton matrix, which is slightly less precise, but it is inexpensive to compute. A detailed explanation of the algorithm can be found in [31].

### 2.5 Outputs

The Dictionary for Secondary Structure of Proteins (DSSP) defined a standardized format of categorizing the secondary structures of a protein [47]. This format proposes eight (8) different classes of secondary structures, based on geometric arrangements and hydrogen bonding patterns, and they are represented by a capital letter of the English Alphabet. The classes are, the *α*-helix (H), 3-helix (G), *π*-helix (I), *β*-strand (E), *β*-bridge (B), *β*-turn (T), bend (S), and random coil (C) for residues which are not in any of the other conformations. The above eight (8) categories are usually grouped into three (3) broader classes that describe the nature of the shape of the specific local segment of the protein. In this paper the 3-class classification is used. This includes the helix (H) conformations that contain the first three categories (H, G, I), the extended (E) conformations, containing the next two categories (E, B), and finally Coil (C) conformations which contain everything else (T, S, C).

### 2.6 Performance measures

To be able to measure how good the predictions of the trained models are, two different metrics were used. First the per residue Q3 accuracy, which measures the number of correctly classified amino acids, divided by the total number of amino acids. Second the Segment Overlap (SOV) score [48] is used to measure the overall quality of the predicted structure by comparing contiguous segments of the same secondary structural class.

### 2.7 Ensembles and filtering methods

Ensemble (ENS) learning, in machine learning, is a method that can be used to enhance the performance of a model [49]. This method works by training multiple models and combining the predictions obtained, instead of just using the predictions of a single model. For our implementation of the PSSP problem, we have trained five different models (with random weight initialization for each model) for each one of the ten folds of the CB513 dataset and for each one of the five folds of the PISCES dataset. Each model produced a prediction/output (H, E, C) for a specific input. The predictions are then compared with the method ‘winner takes all’ and the class with the most appearances is chosen as the final class of the specific input [50]. In case of a tie between some of the classes, an arbitrary class from those tied is selected as the final class. The use of ensembles can remove random errors from the models, which may result in better overall predictions.

Post-processing filtering is another way of improving the predictions of a model. A filtering technique can either be applying another learning algorithm on the existing predictions [35], or may use some predefined (empirical) external rules (ER) on the predictions. Post-processing techniques are beneficial for the PSSP problem because they enhance the quality of predictions by eliminating conformations that are physicochemically unlikely, such as single-helical residues that cannot form a stable helix. In previous work by our group, we have illustrated that filtering approaches can significantly improve protein secondary structure prediction [35], leading to improved accuracy (especially with regards to SOV). In this work, both filtering types were utilized in order to observe the impact of each filtering technique.

External rules are based on empirical observations and are specific for the PSSP problem, targeting on improving the SOV score rather than the Q3 accuracy. They are also computationally cheap. The external rules we are using in this paper were derived in [51].

**Fig. 1:**
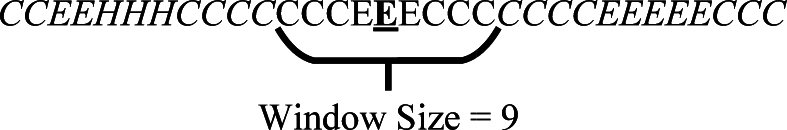
Post-processing Window size illustration.

Applying another learning algorithm to the existing predictions demands the creation of a new training and test set. The new sets are now created using the predicted secondary structures obtained from the CNN using a post-processing window. The window size indicates the number of neighbouring secondary structural states that are going to be used to construct the inputs for post-processing. An example of how the window size works is illustrated in Fig. 1. The window size plays a major role in the accuracy of the predictions. A bigger window size can capture long range connections/interactions between classes resulting in better Q3 accuracy.

In this paper three different filtering algorithms are applied: The Support Vector Machine (SVM) [52], the Random Forest (RF) [53] and Decision Trees (DT) [54]. The SVM classifier was trained using the RBF kernel with a scale kernel coefficient (gamma) and a regularization value (C) of 100. The RF classifier was configured with 100 estimators and a maximum depth of 25, whereas the DT classifier was trained with a maximum depth of 20.

## 3 Results and discussion

As mentioned in Section 2.1 the 10-fold cross validation technique was used for the experiments performed for the CB513 dataset, where nine folds were used for training and one fold was used for validation. Before training any models, several experiments were performed using fold 8 of the CB513 dataset as the validation set while training on the remaining nine folds, to fine-tune the hyper-parameters of the CNN to achieve optimal results. Fold 8 was chosen as the validation set for fine-tuning since it exhibited the lowest prediction accuracy among all the folds. The idea was that by improving the prediction accuracy for this fold, there would be a significant enhancement in the performance of the model across all folds. Furthermore, as mentioned in Section 2.4 pooling layers were removed from the CNN architecture after some preliminary experiments showed that CNNs with pooling layers performed worse than CNNs without pooling layers (Q3 accuracy with pooling layers ≈ 75.5%, Q3 accuracy without pooling layers ≈ 78.5%, using fold 0 of CB513).

After the hyper-parameters were selected, five CNN models for each fold were trained, using the SHN method. Except from the validation score for each model, the CASP13 dataset was used as an independent testing set. For each fold, the predictions of the five individual models were combined with the ‘winner takes all’ ensembles method [50] and various filtering techniques were then applied in order to improve the final results. Table 1 shows the 10-fold cross validation results for CB513 and the average independent test scores for CASP13, for all methods used using a post-processing window size of 19.

**Table 1:** Summary of the results of this work for the 10-fold cross validation of CB513 and the independent test dataset (CASP13) after applying ensembles and various filtering methods. Bold values indicate the best results (see text for details).

Method & Filtering Order	CB513	(Valid.)	CASP13	(Test)
	Q3(%)	SOV	Q3(%)	SOV
CNN 10-Fold Average	79.96	71.76	78.6	70.77
CNN Ensembles (5 models)	80.32	72.44	78.54	70.75
CNN Ens. + ER Filt.	80.4	75.78	78.6	73.04
CNN Ens. + ER + SVM Filt.	85.58	80.3	82.97	77.17
CNN Ens. + SVM Filt.	90.71	86.76	86.95	82.38
CNN Ens. + SVM + ER Filt.	89.92	85.34	86.35	82.83
CNN Ens. + ER + DT Filt.	87.67	81.62	85.67	79.14
CNN Ens. + DT Filt.	93.04	88.59	90.08	85.08
CNN Ens. + DT + ER Filt.	91.89	86.91	88.99	86.05
CNN Ens. + ER + RF Filt.	87.78	81.37	85.72	79.52
CNN Ens. + RF Filt.	**93.65**	**89.63**	**90.54**	86.49
CNN Ens. + RF + ER Filt.	92.66	88.13	89.77	**87.47**

The best results for the CB513 dataset were achieved when the RF filtering technique was applied after the ensembles method, giving 93.65% Q3 accuracy and an SOV score of 89.63. The higher Q3 accuracy for the CASP13 dataset when the system was trained on CB513 is when the ensembles results are filtered with RF at 90.54%, whereas the best SOV score of 87.47 is when ensembles results are filtered with RF and external rules.

For the PISCES dataset the 5-fold cross validation approach was used, as mentioned in Section 2.1. Table 2 shows the 5-fold cross validation results for PISCES and the average independent test scores for CASP13, for all methods used using a post-processing window size of 19.

**Table 2:** Summary of the results of this work for the 5-fold cross validation of PISCES and the independent test dataset (CASP13) after applying ensembles and various filtering methods. Bold values indicate the best results – see text for details.

Method & Filtering Order	PISCES	(Valid.)	CASP13	(Test)
	Q3(%)	SOV	Q3(%)	SOV
CNN 5-Fold Average	81.45	75.91	78.83	71.62
CNN Ensembles (5 models)	81.59	76.31	78.84	72.18
CNN Ens. + ER Filt.	81.67	78.80	79.12	74.27
CNN Ens. + ER + DT Filt.	83.07	79.67	86.29	80.69
CNN Ens. + DT Filt.	85.71	82.14	90.30	86.07
CNN Ens. + DT + ER Filt.	85.22	82.65	89.24	87.08
CNN Ens. + ER + RF Filt.	83.22	79.89	86.32	80.76
CNN Ens. + RF Filt.	**87.13**	**84.28**	**90.69**	87.54
CNN Ens. + RF + ER Filt.	86.69	84.25	89.88	**87.94**

The best results for the PISCES dataset were obtained again when the RF filtering technique was used after the ensembles method, giving a Q3 accuracy of 87.13% and an SOV score of 84.28. The best Q3 accuracy for the CASP13 dataset when the system was trained on PISCES dataset is when the ensembles results are filtered with random forest at 90.69%, whereas the best SOV score of 87.94 was achieved when ensembles results are filtered with RF and external rules.

As we can observe from Table 1, Table 2, the use of ensembles altered only slightly the Q3 accuracies and the SOV scores. How the Q3 accuracy and SOV score fluctuate depends on the variance of the predictions between the trained models, but since all models were trained with the same hyper-parameters the variance between these models was small, thus the impact of using the ensembles method was small as well. Moreover, we can see that applying the external rules after a learning algorithm (SVM, DT, RF) for filtering gives better results than when applying the ER before.

Table 3, Table 4 show in more detail the best results, combination of CNN-SHN + ENS + RF filtering, for CB513 (Table 3), and PISCES (Table 4).

**Table 3:** Q3 accuracy and SOV score for ensembles and Random Forest filtering for each fold of CB513.

Fold	Q3	QH	QE	QC	SOV	SOVH	SOVE	SOVC
0	91.38	93.26	86.84	91.8	86.48	87.78	82.58	84.43
1	94.41	95.11	92.79	94.81	89.8	92.89	88.15	86.5
2	93.33	92.89	91.61	94.56	87.68	89.83	87.56	83.87
3	93.82	95.99	91.78	92.82	87.99	88.73	89.93	84.83
4	94.19	94.74	91.8	94.82	91.93	90.75	90.24	91.76
5	93.57	93.81	91.62	94.62	90.35	88.56	91.5	89.68
6	93.94	94.74	90.69	94.89	90.57	92.1	90.76	90.47
7	93.04	93.34	88.68	95.2	89.16	89.19	88.39	88.34
8	94.17	93.35	91.87	95.99	90.64	86.74	89.26	90.16
9	94.62	94.71	92.73	95.59	91.67	93.36	90.62	91.03

Avg.	93.65	94.19	91.04	94.51	89.63	89.99	88.9	88.11

**Table 4:** Q3 accuracy and SOV score for ensembles and Random Forest filtering for each fold of PISCES.

Fold	Q3	QH	QE	QC	SOV	SOVH	SOVE	SOVC
0	87.04	89.81	82.51	86.71	83.96	84.31	83.77	79.23
1	87.21	89.84	83.41	86.79	84.38	83.37	85.31	79.51
2	87.16	89.88	82.78	87.01	84.55	84.04	84.84	79.76
3	87.02	89.72	83.01	86.64	83.80	83.47	85.09	78.66
4	87.21	89.58	83.16	87.10	84.69	82.97	85.19	78.53

Avg.	87.13	89.77	82.97	86.85	84.28	83.63	84.84	79.14

Additional experiments were performed in order to see the impact the post-processing window size (that is used to create the datasets for the machine learning filtering techniques) has on the predictions. Since the post-processing window size must be an odd number, we performed experiments for all odd-sized windows from 3 to 31. A size of 31 can be considered a limit since the smaller protein in the CASP13 dataset is 31 and we want to keep the test dataset intact in order to be able to make comparisons. Furthermore, the best filtering technique is RF, so the following experiments are based only on this technique.

**Fig. 2:**
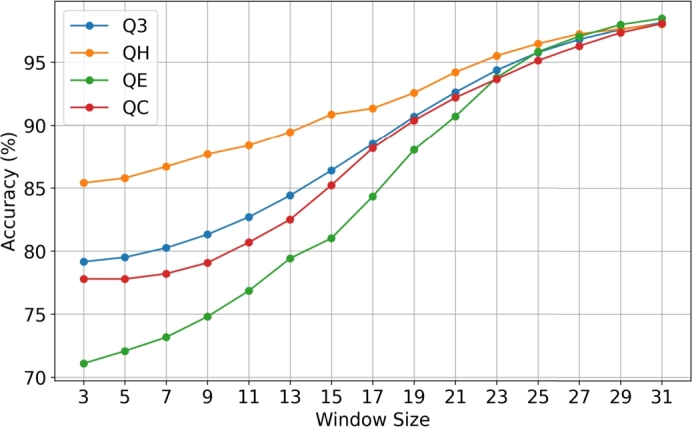
Graph illustration of the average Q3, QH, QE and QC accuracies, based on the post-processing window size, for Random Forest on CASP13 trained with PISCES dataset.

**Fig. 3:**
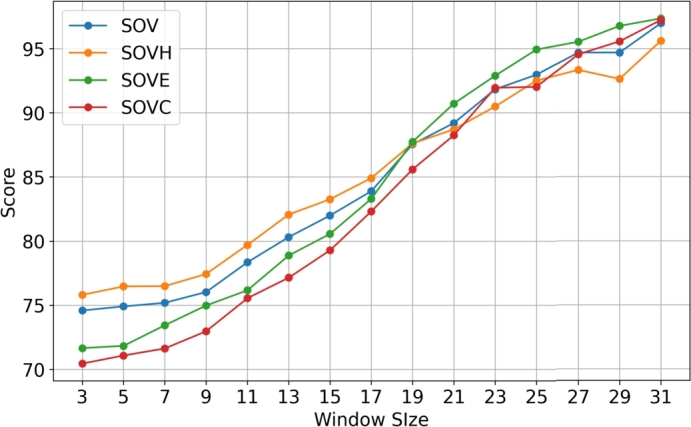
Graph illustration of the average SOV, SOVH, SOVE and SOVC scores, based on window size, for Random Forest on CASP13 trained with PISCES dataset.

According to Fig. 2 we can see that as the post-processing window size increases, the prediction accuracy increases as well. Moreover, we can see that the curves that illustrate the accuracy of each class (QH, QE, QC) and the overall Q3 accuracy converge at around 98%. Finally, we can observe from Table 5 that initially (i.e., for window size = 3) the QE accuracy is the worst with just over 71% but at the end (i.e., for window size = 31) QE is the best predicted class with an accuracy of 98.46%. As mentioned in Section 2.7, a larger post-processing window size may capture longer range connections/interactions between classes resulting in better Q3 accuracy, and this is proved by the results shown in the graph of Fig. 2. The same experiment was used to analyze the SOV score as well. The results are shown in Table 6 and Fig. 3. When using window size from 3 to 17, the SOVE score was the worst but from window size 19 to 31 the SOVE score was the best.

**Table 5:** Average Q3, QH, QE and QC accuracies, based on the post-processing window size, for Random Forest on CASP13 trained with PISCES dataset, using the ProtBert embeddings representation.

	Q3	QH	QE	QC
WINDOW 3	79.15	85.40	71.09	77.79
WINDOW 5	79.50	85.78	72.08	77.78
WINDOW 7	80.25	86.70	73.17	78.20
WINDOW 9	81.32	87.68	74.81	79.07
WINDOW 11	82.70	88.38	76.85	80.69
WINDOW 13	84.40	89.45	79.42	82.50
WINDOW 15	86.39	90.86	81.02	85.22
WINDOW 17	88.51	91.34	84.33	88.17
WINDOW 19	90.69	92.57	88.03	90.39
WINDOW 21	92.62	94.21	90.71	92.20
WINDOW 23	94.37	95.52	93.78	93.67
WINDOW 25	95.78	96.47	95.83	95.14
WINDOW 27	96.79	97.23	97.04	96.28
WINDOW 29	97.58	97.63	97.97	97.33
WINDOW 31	98.12	98.01	98.46	98.05

**Table 6:** Average SOV, SOVH, SOVE and SOVC scores, based on post-processing window size, for Random Forest on CASP13 trained with PISCES dataset, using the ProtBert embeddings representation.

	SOV	SOVH	SOVE	SOVC
WINDOW 3	74.58	75.80	71.65	70.44
WINDOW 5	74.90	76.46	71.82	71.07
WINDOW 7	75.18	76.48	73.43	71.62
WINDOW 9	76.02	77.43	74.97	72.96
WINDOW 11	78.34	79.70	76.16	75.54
WINDOW 13	80.31	82.07	78.87	77.14
WINDOW 15	81.99	83.26	80.56	79.28
WINDOW 17	83.89	84.91	83.29	82.31
WINDOW 19	87.54	87.61	87.74	85.59
WINDOW 21	89.20	88.71	90.72	88.25
WINDOW 23	91.83	90.49	92.89	91.95
WINDOW 25	92.97	92.51	94.93	92.02
WINDOW 27	94.69	93.33	95.53	94.55
WINDOW 29	94.69	92.63	96.77	95.56
WINDOW 31	96.98	95.61	97.35	97.23

The same experiment was performed on the results when the same CNN with the HFO was trained on a different data representation, implemented by Dionysiou et al. [16] and used by Leontiou [33]. It is a two-dimensional (2D) input representation method, where Multiple Sequence Alignment (MSA) profile vectors are placed one under another.

**Fig. 4:**
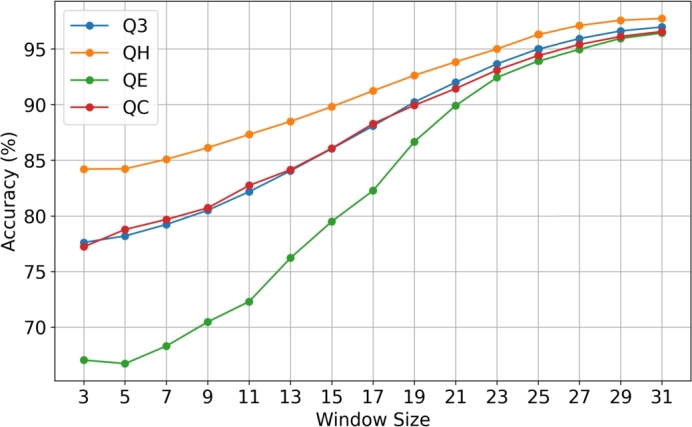
Graph illustration of the average Q3, QH, QE and QC accuracies, based on post-processing window size, for Random Forest on CASP13 trained with PISCES dataset using the data representation by Dionysiou et al. [16].

**Fig. 5:**
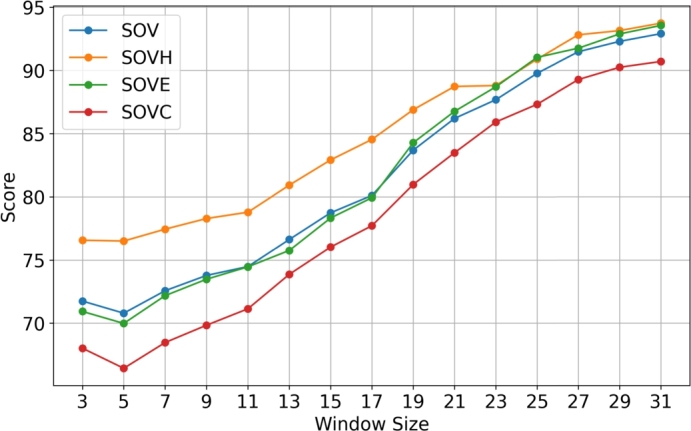
Graph illustration of the average SOV, SOVH, SOVE and SOVC scores, based on window size, for Random Forest on CASP13 trained with PISCES dataset using the data representation by Dionysiou et al. [16].

From Table 7 and Fig. 4 we can observe how the Q3 accuracy varies as we change the post-processing window size using the data representation by Dionysiou et al. [16]. For the smallest window we have a Q3 accuracy of 77.61% and for the biggest window we have a Q3 accuracy of 96.97%. Table 8 and Fig. 5 show how the SOV score increases as the post-processing window size increases as well for the data representation by Dionysiou et al. [16]. For the smallest post-processing window, we have an SOV score of 71.75 and for a window size of thirty-one (31) we have an SOV score of 92.90. Now if we compare the results between the two different data representations, we can draw some conclusions. The Q3 accuracy when the smallest post-processing window size was used (window size 3), was for the data representation that used embeddings 79.15% (Table 5) and for the other data representation [16] 77.61% (Table 7). There is a difference of almost 1.5%, with this difference fluctuating a bit as the post-processing window size increases, but even when the biggest window size was used the difference in Q3 accuracy remains around 1.5%. The same applies for the SOV score as well, where there is a difference of just under 3, but this difference remains as the post-processing window size increases. This shows that the filtering techniques produce a proportional increase on the Q3 accuracy and SOV score irrespective of the data representation that was used to train the CNN.

**Table 7:** Average Q3, QH, QE and QC accuracies, based on post-processing window size, for Random Forest on CASP13 trained with PISCES dataset using the data representation by Dionysiou et al. [16].

	Q3	QH	QE	QC
WINDOW 3	77.61	84.21	67.03	77.22
WINDOW 5	78.20	84.23	66.71	78.78
WINDOW 7	79.23	85.09	68.29	79.68
WINDOW 9	80.51	86.13	70.46	80.72
WINDOW 11	82.18	87.32	72.29	82.74
WINDOW 13	84.05	88.49	76.21	84.15
WINDOW 15	86.04	89.81	79.50	86.07
WINDOW 17	88.08	91.24	82.27	88.29
WINDOW 19	90.23	92.63	86.67	89.94
WINDOW 21	92.00	93.84	89.91	91.44
WINDOW 23	93.66	95.00	92.44	93.10
WINDOW 25	95.00	96.31	93.92	94.41
WINDOW 27	95.93	97.11	94.96	95.40
WINDOW 29	96.62	97.58	95.95	96.12
WINDOW 31	96.97	97.74	96.43	96.57

**Table 8:** Average SOV, SOVH, SOVE and SOVC scores, based on post-processing window size, for Random Forest on CASP13 trained with PISCES dataset using the data representation by Dionysiou et al. [16].

	SOV	SOVH	SOVE	SOVC
WINDOW 3	71.75	76.57	70.93	68.02
WINDOW 5	70.79	76.51	69.98	66.43
WINDOW 7	72.57	77.45	72.18	68.47
WINDOW 9	73.78	78.29	73.48	69.84
WINDOW 11	74.48	78.79	74.46	71.14
WINDOW 13	76.63	80.93	75.76	73.87
WINDOW 15	78.74	82.92	78.33	76.03
WINDOW 17	80.11	84.54	79.93	77.72
WINDOW 19	83.69	86.89	84.28	80.98
WINDOW 21	86.20	88.73	86.76	83.49
WINDOW 23	87.68	88.80	88.70	85.92
WINDOW 25	89.77	90.89	91.03	87.32
WINDOW 27	91.47	92.81	91.75	89.27
WINDOW 29	92.29	93.14	92.88	90.24
WINDOW 31	92.90	93.73	93.55	90.70

### 3.1 A note on protein secondary structure prediction using single-sequences

With the increasing availability of high-quality predicted protein 3D structures, many researchers will argue that protein secondary structure prediction might only serve as a test-bed for evaluating the suitability of new algorithms for predicting protein structural features. Nevertheless, there are research settings where obtaining full protein 3D structures might be unfeasible (e.g., due to limited computational resources) and 1D predictions (e.g., Secondary Structure Prediction) could give useful information for guiding experimental design, or serving as input for downstream computational methods.

We start with the working hypothesis that template-based methods (i.e., using structural information of homologs with a known structure in the PDB) and profile-based methods (i.e., using evolutionary information) would predict secondary structures with performance equivalent to that of AlphaFold2. Such methods require an initial resource-intensive step of sequence database search, therefore it would be interesting to further develop single-sequence protein structure prediction methods to the same levels of accuracy as good alternatives when limited computational resources are available or when extra speed is necessary.

With this in mind, we provide a preliminary comparison of the most basic version of our method against two state-of-the-art servers for predicting protein secondary structures based on single polypeptide sequences: NetSurfP 3.0 [55] and S4PRED [56] (both accessed online between November-December 2024). We use 10 predictors trained on the 10 CB513 folds with the CNN-HFO algorithm and develop a consensus majority vote ensemble (CNN-HFO-CB513) and its post-processed output using simple empirical rules (see Section 2: Materials and Methods). We selected 26 free modelling targets from the CASP15 community experiment [57], as “hard” prediction targets, with none of them baring significant sequence similarity to the dataset used to train the CNN-HFO-CB513 models. As a gold standard, we report the AlphaFold2 predictions for the respective targets, processed with the newest version of the DSSP software (mkdssp version 4.2.2) [47], [58] to yield secondary structure predictions.

The results of this preliminary benchmark are summarized in Table 9. We observe that NetSurfP 3.0 and S4PRED obtain similar levels of accuracy, with the former obtaining superior SOV; our simple CNN-HFO implementations follow in perfomrance metrics. Given that NetSurfP 3.0 and S4PRED were both trained on datasets with more than 10,000 sequences and that the cross-validation results of the CNN-HFO approach on the much larger PISCES dataset resulted in higher Q3 and SOV values compared to models trained on CB513 (approx. 1.5% Q3 and 4% SOV), we can postulate that the CNN-HFO performance is comparable to these currently top-performing single-sequence methods, but further work is needed for a conclusive result. Importantly, all single-sequence predictors significantly lag in performance compared to AlphaFold2. This suggests that further room (and need) for improvement exists in the 3-state protein secondary structure prediction field (and in the harder 8-state PSSP).

**Table 9:** Secondary structure prediction with single sequence information for the hard (free modelling) targets of the CASP15 dataset.

	Q3	SOV
NetSurfP 3.0	80.55	69.43
S4PRED	80.31	57.42
CNN-HFO-CB513	75.29	54.64
CNN-HFO-CB513 + ER filt	75.37	60.54

**AlphaFold2**	**88.23**	**78.53**

### 3.2 Case study: secondary structure prediction of select metamorphic proteins

Apart from the speed-up obtained during the training phase by use of the second order HFO method, models trained in our work are quite fast during the inference (prediction) phase. This is due to the fact that sequence embeddings are much faster to compute from pre-trained protein large language models compared to computing deep multiple sequence alignments for homologs obtained from vast sequence datasets. This advantage enables us to easily analyse the predictions produced by our method on particular proteins of interest.

**Fig. 6:**
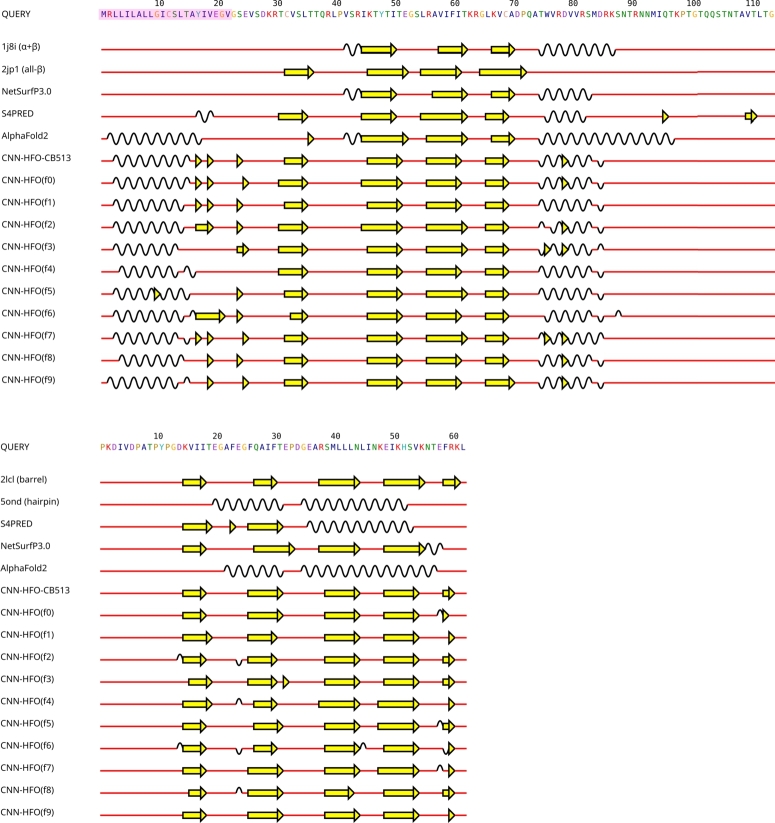
Observed and predicted secondary structures for two metamorphic proteins. **Top:** Human lymphotactin and **Bottom:** C-terminal domain (residues 101-162) of the transcription antitermination protein RfaH from *E. coli*. In both panels, the amino acid sequence (QUERY) is followed by secondary structures based on two experimentally determined structures representing their alternative folds followed by predicted secondary structures by NetSurfP 3.0 and CNN-HFO, as well as the secondary structure assignment (using DSSP) for the respective predicted 3D structures from the AlphaFold database [65] (accessed December 2024). The shaded sequence segment in the top panel signifies the absence of structural information in the PDB entries for the N-terminal segment (1-22) of lymphotactin. CNN-HFO(cons) displays the majority vote ensemble of ten independent predictions – displayed as CNN-HFO(f0)-CNN-HFO(f9) – for classifiers trained on the CB513 cross-validation training sets. No post-processing (other than the ensemble) was applied to CNN-HFO predictions. Helices are depicted as black waves, extended *β*-strands as yellow arrows, and coil as red straight lines.

One interesting case consists of metamorphic proteins, which are known to reversibly switch between different (unrelated) folds under physiological conditions. Fig. 6 displays secondary structural information visualized (using the 2dSS web server [59]) for two well-studied cases of metamorphic proteins, namely:

• the human C-class chemokine lymphotactin (UniProt ID: P47992), which has been reported to switch between a monomeric form adopting the typical α+β chemokine fold and a homodimeric all-*β* fold [60], [61].
• the two-domain transcription antitermination protein RfaH from *Escherichia coli* (UniProt ID: P0AFW0), whose C-terminal “Kyrpides, Ouzounis, Woose” (KOW) domain [62] has been shown to switch between a *β*-barrel and an *α*-hairpin fold [63], [64].

The full sequences for these proteins were subject to secondary structure prediction with NetSurfP 3.0 [55], S4PRED [56] and the majority vote ensemble of CNN-HFO models trained on the 10 folds of the CB513 dataset. In the case of lymphotactin we observe that all prediction methods (including AlphFold2) tend to produce predictions that mix characteristics of the two metamorphic states. On the other hand, results for the RfaH C-terminal domain exhibit a different pattern: AlphaFold2 prediction coincides with the helical hairpin conformation and CNN-HFO with the *β*-barrel arrangement (with NetSurfP 3.0 predicting approximately the latter), while S4PRED mixes features from the two conformational states. A more thorough analysis is warranted to shed light on whether internal parameters (e.g., prediction probabilities for different SS states by NetSurfP 3.0/CNN-HFO, pLDDT values for AlphaFold2) or suboptimal predictions (e.g., lower-ranked AlphaFold2 models) of the different predictors could highlight the potential of these proteins (or even specific regions/domains) to switch between unrelated folds.

## 4 Conclusion

The main purpose of this paper was to use embeddings that were extracted from language models as inputs to a CNN that uses a second order optimization algorithm, the SHN method, in order to train models that predict the Secondary Structure of Proteins (PSSP), given its primary structure. To this effect, our work can be classified in the post-AlphaFold [26] category of the deep learning for PSSP techniques, according to a recent classification by Ismi et al. [28], in which protein language models are exploited as input features.

The best results from this study were 93.65% Q3 accuracy and 89.63 SOV score for the CB513 dataset (Table 1), while for the PISCES dataset, the results were 87.13% Q3 accuracy and 84.28 SOV score. Furthermore, the best results concerning the test dataset (CASP13) were 98.12% Q3 accuracy and 96.98 SOV score (Section 3, Table 5 & Table 6). Based on the results we can draw the following conclusions. The use of embeddings as inputs to the CNN gave results equally good or even slightly better than in the case when MSA profile vectors placed one under another [16] are used [33]. This makes the use of embeddings more convenient for multiple reasons. While existing solutions in Protein Bioinformatics usually have to search for evolutionary related proteins in exponentially growing databases, language models offer a potential alternative to this increasingly time-consuming database search as they extract features directly from single protein sequences. Our results agree with recent work using embeddings as encoding for solving relevant problems from protein bioinformatics [66], [67], [68], [69]. Moreover, the performance of existing MSA-based solutions decreases if not enough related sequences can be found, e.g., the quality of predicted protein structures correlates strongly with the number of effective sequences found in today's databases. Additionally, some proteins are intrinsically hard to align (e.g., intrinsically disordered proteins or proteins which do not have any related sequences, such as synthetic sequences). On the other hand, embeddings require a considerable amount of storage space.

Finally, filtering techniques were the ones that boosted the results. As we can observe, the order that the filtering techniques were applied played a significant role in the outcome. However, the most important aspect of the post processing methods was the size of the post-processing window (Section 2.7). As the post-processing window size was increasing, the results were getting better as well. This concludes that a larger post-processing window can better capture long range connections between secondary structure elements, resulting in higher Q3 accuracy and SOV.

## CRediT authorship contribution statement

**Sotiris Chatzimiltis:** Formal analysis, Investigation, Methodology, Software, Validation, Visualization, Writing – original draft, Writing – review & editing. **Michalis Agathocleous:** Formal analysis, Investigation, Methodology, Project administration, Software, Validation, Visualization, Writing – original draft, Writing – review & editing. **Vasilis J. Promponas:** Conceptualization, Data curation, Formal analysis, Investigation, Methodology, Project administration, Resources, Software, Supervision, Validation, Visualization, Writing – original draft, Writing – review & editing. **Chris Christodoulou:** Conceptualization, Data curation, Formal analysis, Investigation, Methodology, Project administration, Resources, Software, Supervision, Validation, Visualization, Writing – original draft, Writing – review & editing.

## Declaration of Competing Interest

The authors wish to declare that Vasilis J. Promponas is a member of the editorial board of Computational and Structural Biotechnology Journal. No other potential conflicts of interest exist.
